# Hand Hygiene and Mask-Wearing Practices during COVID-19 among Healthcare Workers: Misinformation as a Predictor

**DOI:** 10.4269/ajtmh.21-0463

**Published:** 2021-10-22

**Authors:** Senhu Wang, Lambert Zixin Li, Natasha van Antwerpen, Sutrisno Suparman, Mergy Gayatri, Ningrum Paramita Sari, Stephen X. Zhang

**Affiliations:** ^1^ Department of Sociology, National University of Singapore, Singapore;; ^2^ Graduate School of Business, Stanford University, Stanford, California;; ^3^ School of Psychology, University of Adelaide, Adelaide, South Australia, Australia;; ^4^ Faculty of Medicine, Universitas Brawijaya, Malang, East Java, Indonesia,; ^5^ Faculty of the Professions, University of Adelaide, Adelaide, Australia

## Abstract

Poor public health information is a hurdle in infectious disease control. The study aims to examine whether healthcare workers adhere to hand hygiene and mask-wearing guidelines during the COVID-19 pandemic and to explore their exposure to misinformation about the pandemic as a predictor. A cross-sectional survey was sent to 518 healthcare workers across Indonesia, the fourth largest nation in the world, in September 2020 during the COVID-19 pandemic. The respondents reported whether they adhered to the guidelines of hand hygiene and mask wearing and whether they believed in four pieces of misinformation about the origin, severity, contagion, and prevention of COVID-19. The association between misinformation and hand hygiene and mask wearing was tested with logistic regression models controlling for demographic and health-related covariates. Approximately 25% of healthcare workers did not always adhere to hand hygiene guidelines and approximately 5% did not adhere to mask-wearing guidelines. There are significant associations between all four pieces of misinformation and hand hygiene and mask wearing. It is important to improve public health information about COVID-19, which may hold key to healthcare workers’ hand hygiene and mask wearing and to protect their health and patients’ safety.

## INTRODUCTION

As COVID-19 has become a global pandemic, and scholars call for the prevention of its spread using social and behavioral sciences.^1^^–^^3^ The WHO and the CDC have repeatedly emphasize the importance of preventive behaviors such as handwashing and mask wearing in cutting down the spread of the virus.^4^^,^^5^ However, the public’s adherence to these guidelines remain low in many places.^6^ Among all professions, healthcare workers have a heightened responsibility to adhere to the hand hygiene and mask wearing guidelines. On one hand, preventive behaviors are necessary to the healthcare workers’ mental and physical health.^7^^–^^9^ Healthcare workers play a vital role in taking care of COVID-19 patients and other patients with scarce hospital resources during the pandemic period. Fulfilling these duties depends on their own infection control and health.^10^ On the other hand, given the long incubation period of COVID-19, infection of COVID-19 by healthcare workers puts patients’ health and safety at risks.^1^ Despite the repeated calls to increase healthcare workers’ awareness of, access to, and abidance by hand hygiene and mask wearing guidelines, previous research on this topic relies on literature review or expert opinions.^11^^,^^12^ There lacks empirical evidence on whether healthcare workers indeed adhere to these guidelines during the ongoing pandemic. Therefore, the first goal of the study is to explore the compliance rates of hand hygiene and mask-wearing guidelines among healthcare workers during the COVID-19 pandemic.

Adherence to handwashing and mask wearing depends on various social and psychological factors, among which the misinformation about COVID-19 has been identified as a consistent predictor in previous research on the general populations.^13^^–^^15^ These studies examine misinformation about various facets of COVID-19, including whether it is engineered by human in a laboratory (origin), whether it causes severe health risks (severity), whether it can be spread through 5G network (contagion), and whether it can be prevented by eating garlic (prevention).

Although previous studies offer evidence on the link between the beliefs in misinformation and preventive behaviors,^13^^–^^15^ we know little about whether the association is also present among healthcare workers. Although healthcare workers have the specialist knowledge to be less susceptive to misinformation, they may also hold misbeliefs about COVID-19 for several reasons. First, the psychological roots of rumors and conspiracy theories are so entrenched that even professionals sometimes fall into the traps. Research shows that when exposed to misinformation, affective responses lead to information avoidance and distorted information processing, and these psychological mechanisms are common in human decision-making under uncertainties.^1^ Second, the COVID-19 pandemic evolves rapidly and causes great mental distress.^3^^,^^16^ There is quick dissemination of critical information and much unverified and conflicting information on social media, which could have led to information overload, especially among healthcare workers who lacked work–family balance and supportive social networks.^17^^–^^19^ Third, the infrastructure for public health communications could be less developed in the developing countries.^20^^,^^21^ Information insufficiency, especially paucity of updated information verified by public health authorities, may lead the healthcare workers in less developed countries or regions to believe in misinformation.^22^ In other words, even if some healthcare workers are not prone to misinformation, there are delays and geographic disparities in receiving and absorbing accurate health information.^23^^,^^24^ For these reasons, we hypothesize that the link between misinformation and noncompliance with hand hygiene and mask-wearing guidelines is also present among healthcare workers.

The setting of the study is the healthcare sector in Indonesia, which is the fourth largest nation in the world with a population of more than 260 million.^25^ The country was heavily hit by the COVID-19 pandemic, with > 1.6 million cases and 44,594 deaths as of April 26, 2021, at the time of submission of this article.^26^ Previous research documents high prevalence rates of misinformation among the general public in the country and identifies key operational challenges for its healthcare systems.^2^^,^^27^ However, to our knowledge, there has not been no study on misinformation and preventive behaviors including handwashing and mask wearing among the healthcare workers in the country, calling for more empirical evidence on this important population. Understanding of healthcare workers’ health behaviors holds significant implications for their health and the patients’ safety.^28^^–^^30^

## METHODS

### Data and sample.

The data for this study were collected by an online survey on September 26, 2020, when there was a total of 271,339 confirmed cases of COVID-19 and 10,308 deaths in Indonesia.^31^ The study site is State Polytechnic of Health Malang Indonesia. The study population is healthcare workers in Indonesia. The participants of the survey included healthcare workers from more than 200 Indonesian cities who were attending an online seminar conducted on September 26, 2020. Convenience sampling was used, but the respondents were diverse in socioeconomic characteristics (as described in Table 1) to represent the study population. The online survey reached 786 healthcare workers, and we received 518 valid responses (response rate of 65.9%). Moreover, none of the participants were involved in any of the planning, execution, and reporting stages of the study.

**Table 1:** Sample characteristics

	M, %	Range
Frequency of washing hands, M (SD)	5.93 (1.34)	1–7
Frequency of wearing a mask outside the home, M (SD)	6.63 (0.81)	1–7
Age, M (SD)	29.24 (7.59)	18–55
Ethnicity, %		
Malay	5.79	
Batak	4.83	
Java	58.69	
Sunda	9.27	
Others	21.43	
Education levels, %		
High school	12.55	
Diploma	42.08	
Bachelor’s	20.85	
Honors	11.39	
Master	13.13	
Professions, %		
Healthcare practitioners	56.76	
Healthcare lecturers	10.42	
Healthcare interns and students	23.36	
Other	9.46	
Whether have chronic diseases, %		
Yes	6.76	
No	93.24	
Whether have had flulike symptoms in past 6 months, %		
Yes	19.69	
No	80.31	
Number of respondents	518	

M = Mean; SD = standard deviation.

### Measures.

#### Dependent variables.

In this study, there are two dependent variables to measure preventive health behavior: frequency of washing hands and frequency of wearing a mask. Specifically, the respondents were asked about the frequency of washing hands for 20 seconds or longer after touching objects outside the home, and the frequency of wearing a mask outside the home on a scale ranging from 1 (never) to 7 (anytime). Given that both variables are highly skewed, we combine the categories from 1 to 5, and the categories from 6 to 7 to construct the binary variables to measure whether the healthcare workers carried out frequent preventive health behavior (90% of the time or above). A robustness check analyzing the frequencies of preventive health behavior as metrical variables yields consistent results.

#### Independent variables.

The independent variable is COVID-19 misinformation beliefs, which are measured by four separate statements. Specifically, the respondents reported the extent to which they agree with the following four statements on a scale ranging from 1 (completely disagree) and 5 (completely agree): 1) “Coronavirus was deliberately engineered,” 2) “Coronavirus is no worse than a common cold,” 3) “The 5G mobile network is spreading coronavirus,” 4) “Eating garlic will prevent you from catching the coronavirus.” Due to small sample sizes, misinformation beliefs are recoded to have three categories (“completely agree or agree,” “neither agree or disagree,” “disagree or completely disagree”) in multivariate regression models.

#### Control variables.

This study also controlled for several sociodemographic characteristics of the healthcare workers, including their age, ethnicity, education levels, professions, whether they had chronic diseases and whether they had had flulike symptoms in the past 6 months. These variables are included because they are relevant to misinformation and preventive health behaviors according to previous studies.^13^^,^^14^ More details about categories of each variable and its distribution are in Table 1.

### Analytic strategy.

This study first used descriptive analyses to describe the patterns and distributions of hand hygiene and mask-wearing compliance and those of four COVID-19 misinformation beliefs. Next, this study conducted multivariate analyses to examine the associations between healthcare workers’ COVID-19 misinformation beliefs and preventive health behaviors. As the main outcome variables were binary, we used binary logistic regression models to examine the association between each of the four misinformation beliefs and frequent preventive health behaviors. To ensure the robustness of the results, we treated both dependent variables as metrical variables and use ordinary least squares (OLS) regression to capture greater variation of preventive health behavior frequencies. All analyses were performed using STATA 15. The data were secured in an encrypted computer of the first author.

### Ethics.

The survey was approved by the Ethics Committee of State Polytechnic of Health Malang Indonesia (#931/KEPK-POLKESMA/2020). Participation in this survey was voluntary, and participants could opt-out at any time.

## RESULTS

### Descriptive statistics.

**Figure 1.**
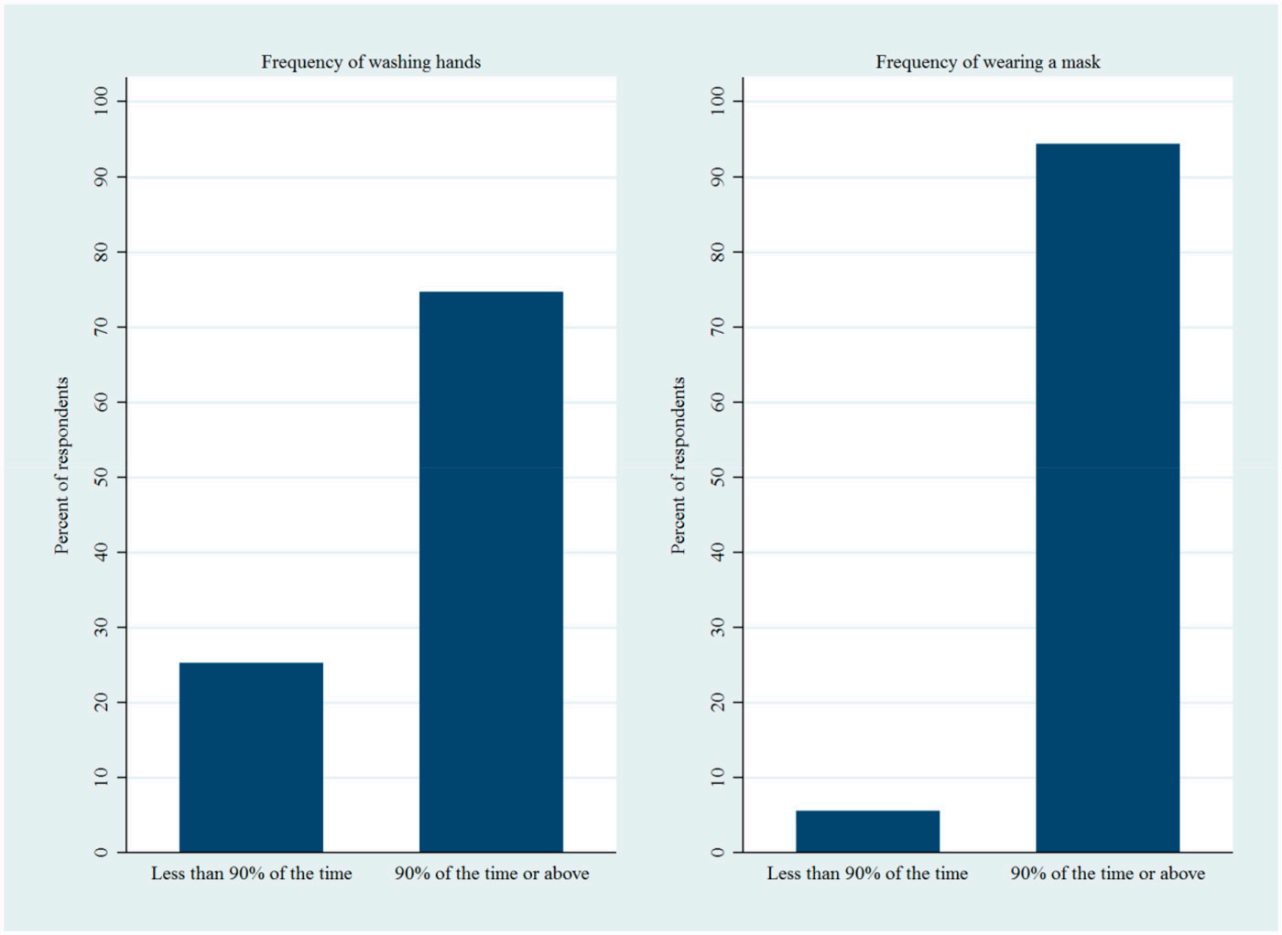
Descriptive statistics for frequencies of handwashing and mask wearing. This figure appears in color at www.ajtmh.org.

Figure 1 shows that ∼75% of the healthcare workers indicated they washed their hands after touching objectives outside the home frequently (≥ 90% of the time) as opposed ∼25% of the respondents who washed their hands less frequently. In contrast, ∼95% of the healthcare workers frequently (at least 90% of the time) wore a mask outside the home, and only 5% of them wore a mask less frequently. Overall, this suggests that although most of the healthcare workers comply with both standard heath practices, mask wearing is a more commonly adopted health practice among the healthcare workers than hand washing.

**Figure 2.**
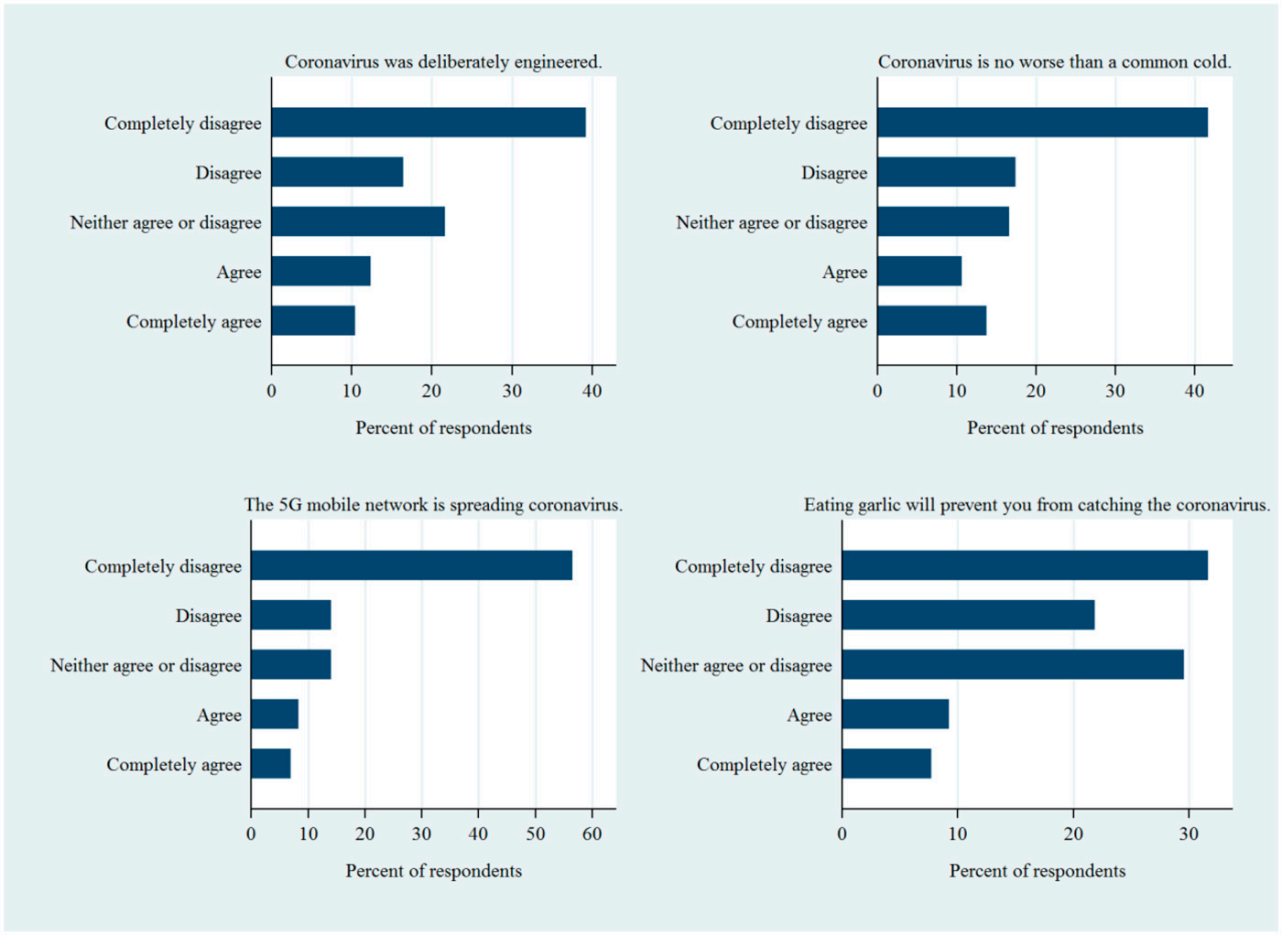
Descriptive statistics for COVID misinformation beliefs. This figure appears in color at www.ajtmh.org.

Figure 2 shows the distributions of four common COVID misinformation beliefs. In terms of whether coronavirus was deliberately engineered, nearly 40% of respondents tended to completely disagree with the statement. This stands in contrast with ∼10% of respondent who agreed or completely agreed with it. The pattern is similar to statements claiming that coronavirus is no worse than a common cold. In terms of whether the 5G mobile network can spread coronavirus, respondents showed a clear attitude and more than half of them completely disagreed with the statement. Finally, in terms of whether eating garlic can prevent coronavirus, although most respondents held an opposing attitude, nearly 30% of respondent could not judge this statement. Such findings suggest that even healthcare workers did not have a clear understanding on this aspect.

### Logistic regression models.

Table 2 shows several logistic regression models and the odds ratios of misinformation beliefs and their confidence intervals. In Panel A, Model 1 shows that in terms of whether coronavirus was deliberately engineered, the respondents who indicated they agreed or neither agreed nor disagreed with this statement were significantly less likely (odds ratios = 0.42 and 0.51) to wash hands frequently after touching objects outside the home than those who did not agree. Similarly, Model 2 shows that respondents who agreed who or neither agreed nor disagreed with the statement that coronavirus is no worse than a common cold were also significantly less likely (odds ratios = 0.54 and 0.53) to frequently wash their hands than their counterparts who did not agree. In terms of whether 5G network is spreading the coronavirus and whether eating garlic can prevent the coronavirus, Models 3 and 4 show that respondents who agreed with these statements were significantly less likely (odds ratios = 0.29 and 0.49) to wash their hands frequently than their counterparts who did not agree.

**Table 2:** Logistic regression models examining the effects of COVID-19 misinformation beliefs on frequent handwashing and frequent mask wearing outside the home.

	Model 1	Model 2	Model 3	Model 4
Misinformation beliefs (Ref. = completely agree or agree)	COVID engineered	COVID is like a common cold	The 5G network spreads COVID	Eating garlic prevents COVID
Neither agree nor disagree	0.42**	0.54*	0.73	0.80
	(0.25–0.71)	(0.31–0.95)	(0.40–1.34)	(0.49–1.31)
Disagree or completely disagree	0.51*	0.53*	0.29***	0.49*
	(0.31–0.86)	(0.33–0.87)	(0.17–0.50)	(0.28–0.86)
Observations	518	518	518	518
Pseudo *R*^2^	0.09	0.09	0.12	0.09

All models control for age, ethnicity, educational level, profession, whether participants had chronic diseases, and whether they had flulike symptoms in the past 6 months. Odds ratios are reported. Confidence intervals are in parentheses.

*** *P* < 0.001; ** *P* < 0.01; * *P* < 0.05 (two-tailed tests).

In Table 3 shows that in terms of whether coronavirus was deliberately engineered (odds ratios = 0.24 and 0.18) and whether coronavirus is no worse than a common cold (odds ratios = 0.26 and 0.20), the healthcare workers who agreed or who neither agreed nor disagreed with these statements were significantly less likely to wear a mask frequently outside the home than those who do not agree. Similarly, in terms of whether 5G network is spreading the coronavirus (odds ratios = 0.10) and whether eating garlic can prevent the coronavirus (odds ratios = 0.14), Models 3 and 4 show that respondents who agreed with these statements were significantly less likely to wear a mask frequently outside the home than their counterparts who did not agree. Further a robustness check (see Table A1 in Supplemental Appendix) analyzing the frequencies of preventive health behavior as metric variables yielded consistent results.

**Table 3:** Logistic regression models examining the effects of COVID-19 misinformation beliefs on frequent mask wearing outside the home

	Model 1	Model 2	Model 3	Model 4
Misinformation beliefs (Ref. = completely agree or agree)	COVID engineered	COVID is like a common cold	The 5G network spreads COVID	Eating garlic prevents COVID
Neither agree or disagree	0.24**	0.26*	0.56	0.67
	(0.08–0.70)	(0.09–0.78)	(0.16–1.97)	(0.21–2.09)
Disagree or completely disagree	0.18**	0.20**	0.10***	0.14***
	(0.06–0.53)	(0.08–0.55)	(0.04–0.26)	(0.05–0.38)
Observations	518	518	518	518
Pseudo *R*^2^	0.09	0.08	0.12	0.11

All models control for age, ethnicity, educational level, profession, whether participants had chronic diseases, and whether they had flulike symptoms in the past 6 months. Odds ratios are reported. Confidence intervals are in parentheses.

*** *P* < 0.001; ** *P* < 0.01; * *P* < 0.05 (two-tailed tests).

## DISCUSSION

In a sample of 518 Indonesian healthcare workers, we found that 1) the noncompliance rate of hand hygiene guidelines was approximately 25%; 2) the noncompliance rate of mask-wearing guidelines was approximately 5%; 3) noncompliance with both hand hygiene and mask wearing were significantly associated with misinformation about COVID-19.

Previous studies have generally found higher noncompliance rates of handwashing and mask-wearing guidelines during the same period in other populations. One study found the “poor behaviors” of hand hygiene and mask wearing to be 57.95% and 48.40%, respectively, among primary school students in Wuhan, China, in February 2020.^6^ Another study found lower noncompliance rates of hand hygiene (38.1%) and mask wearing (44.3%) among Iranian adults (age 16 years or older) from March to April 2020.^31^ A study based in Indonesia found a 43.1% noncompliance rate with hand hygiene guidelines (not always washing hands) among Muslims in May 2020.^32^ In contrast to past studies,^6^^,^^12^ the much lower noncompliance rates of handwashing (25%) and mask wearing (5%) among Indonesian healthcare workers in September 2020 in our study may be explained by their better professional training or higher occupational risks of contagion.^13^ However, approximately one-fourth (25%) of Indonesian healthcare workers in our sample did not fully comply with the hand hygiene guidelines, compared with lower noncompliance rate of mask-wearing guidelines (5%), which is concerning given the vital role of healthcare workers’ hand hygiene in ensuring patient safety while fighting the COVID-19 pandemic.^10^

Moreover, we found that noncompliance with hand hygiene and mask wearing were both associated with misinformation about COVID-19 among Indonesian healthcare workers. These results are in line with previous research on the general populations, which consistently found associations between misinformation and COVID-19 preventive behaviors.^6^^,^^13^^,^^14^^,^^33^ Our results suggested that misinformation may similarly affect medical professionals during a public health emergency, which challenged the lay assumption that healthcare workers are not prone to the misbeliefs due to their medical knowledge or professional trainings.^13^ Furthermore, our study extended past studies^27^^,^^34^ by examining four types of misinformation, including misinformation about the origin, severity, contagion, and prevention of COVID-19. We found that all four types of misinformation were each significantly associated with noncompliance with hand hygiene and mask-wearing guidelines. Drawing from these new findings, public health policies need to address all four types of misinformation in designing targeted interventions.

There are several limitations of this study. First, the cross-sectional design limits our ability to make causal inference. For example, the associations between misinformation and preventive behaviors may be confounded by unobserved respondent characteristics such as personality or suffer common-variance bias due to our reliance on self-reported measures. Future research could use a longitudinal or experimental design to better identify the causal effects of misinformation. Second, although our sample is diverse in terms of sociodemographic characteristics, like most studies on healthcare workers during a pandemic,^1^^,^^10^ we did not use probability sampling to draw a representative sample of Indonesian healthcare workers. Future studies can replicate our exploratory findings using a probability sample of Indonesian healthcare workers or generalize our findings to other countries. Finally, healthcare workers are a heterogenous group consisting of people with different demographic and socioeconomic characteristics. Future research could explore how the effects of misinformation among healthcare workers vary with ethnicity, migration status, residential areas, socioeconomic status social networks, and health condition, for example.^35^

Interventions to reduce COVID-19–related misinformation in the general population can be applied to healthcare workers to increase their preventive behaviors. Information campaigns sponsored by public health authorities and their media partners are a common strategy to curtail health-related misinformation.^36^ In addition, scalable psychological nudging has been shown to decrease misinformation. For example, an experiment found that a simple reminder for judging the accuracy of a non-COVID-19–related headline decreased the sharing of misinformation about COVID-19.^34^ Moreover, given the central role of social networks in spreading misinformation,^37^ it is important for hospital administrators to offer regular trainings and resources to ward-level clinical leaders,^1^ so that they can share the accurate and updated information to other healthcare workers, patients, and the community. Finally, public health infrastructures such as handwashing facilities and personal protective equipment supply must be accessible to increase preventive behaviors even after misinformation is reduced.^1^

## CONCLUSIONS

During the COVID-19 pandemic, most Indonesian healthcare workers adhered to the mask wearing guidelines but approximately one-fourth of them did not fully comply with the handwashing guidelines. Noncompliance with preventive behaviors were associated with their misinformation about the origin, severity, contagion, and prevention of COVID-19. Interventions to reduce misinformation during a pandemic are necessary to improve healthcare workers’ health and well-being and to protect patients’ safety, especially in low- and middle-income countries.

## Supplemental Material


Supplemental materials

## References

[1] Powell-Jackson T King JJC Makungu C Spieker N Woodd S Risha P Goodman C , 2020. Infection prevention and control compliance in Tanzanian outpatient facilities: a cross-sectional study with implications for the control of COVID-19. Lancet Glob Health 8: e780–e789. 32389195 10.1016/S2214-109X(20)30222-9 PMC7202838

[2] Djalante R 2020. Review and analysis of current responses to COVID-19 in Indonesia: period of January to March 2020. Prog Disaster Sci 6: 100091 34171011 10.1016/j.pdisas.2020.100091 PMC7149002

[3] Li LZ Wang S , 2020. Prevalence and predictors of general psychiatric disorders and loneliness during COVID-19 in the United Kingdom. Psychiatry Res 291: 113267. 32623266 10.1016/j.psychres.2020.113267 PMC7326403

[4] World Health Organization , 2020. Advice on the use of masks in the community, during home care, and in health care settings in the context of COVID-19: interim guidance, 19 March 2020. Available at: https://apps.who.int/iris/handle/10665/331493. Accessed May 18, 2021.

[5] Centers for Disease Control and Prevention , 2020. Hand hygiene recommendations, guidance for healthcare providers about hand hygiene and COVID‐19. Available at: https://www.cdc.gov/coronavirus/2019-ncov/hcp/hand-hygiene.html. Accessed May 18, 2021.

[6] Chen X Ran L Liu Q Hu Q Du X Tan X , 2020. Hand hygiene, mask-wearing behaviors and its associated factors during the COVID-19 epidemic: a cross-sectional study among primary school students in Wuhan, China. Int J Environ Res Public Health 17. doi: 10.3390/ijerph17082893. PMC7215913 32331344

[7] Wang S Li LZ Zhang J Rehkopf DH , Leisure time activities and biomarkers of chronic stress: the mediating roles of alcohol consumption and smoking. Scand J Public Health (In press). doi: 10.1177/1403494820987461. 33570003

[8] Lutfiyya MN Chang LF Lipsky MS , 2012. A cross-sectional study of US rural adults’ consumption of fruits and vegetables: do they consume at least five servings daily? BMC Public Health 12: 280 22490063 10.1186/1471-2458-12-280 PMC3365871

[9] Wang S Li S , 2019. Exploring generational differences of British ethnic minorities in smoking behavior, frequency of alcohol consumption, and dietary style. Int J Environ Res Public Health 16: 2241. 10.3390/ijerph16122241 PMC6616626 31242661

[10] Zhang SX Liu J Afshar Jahanshahi A Nawaser K Yousefi A Li J Sun S , 2020. At the height of the storm: healthcare staff’s health conditions and job satisfaction and their associated predictors during the epidemic peak of COVID-19. Brain Behav Immun 87: 144–146. 32387345 10.1016/j.bbi.2020.05.010 PMC7199703

[11] Sommerstein R 2020. Risk of SARS-CoV-2 transmission by aerosols, the rational use of masks, and protection of healthcare workers from COVID-19. Antimicrob Resist Infect Control 9: 100. 32631450 10.1186/s13756-020-00763-0 PMC7336106

[12] Gon G Dancer S Dreibelbis R Graham WJ Kilpatrick C , 2020. Reducing hand recontamination of healthcare workers during COVID-19. Infect Control Hosp Epidemiol 41: 870–871. 32248864 10.1017/ice.2020.111 PMC7167489

[13] Freeman D , 2020. Coronavirus conspiracy beliefs, mistrust, and compliance with government guidelines in England. *Psychol Med* (In press). doi: 10.1017/S0033291720001890. PMC7264452 32436485

[14] Romer D Jamieson KH , 2020. Conspiracy theories as barriers to controlling the spread of COVID-19 in the U.S. Soc Sci Med 263: 113356. 32967786 10.1016/j.socscimed.2020.113356 PMC7502362

[15] Earnshaw VA Eaton LA Kalichman SC Brousseau NM Hill EC Fox AB , 2020. COVID-19 conspiracy beliefs, health behaviors, and policy support. Transl Behav Med 10: 850–856. 32910819 10.1093/tbm/ibaa090 PMC7499784

[16] Gong S Li LZ Wang S , 2021. Youth mental health before and after the control of the coronavirus disease 2019: a nationally representative cohort study of Chinese college students. J Affect Disord Reports 3: 100066. 10.1016/j.jadr.2020.100066 PMC8995102 35434689

[17] Kalliath T Brough P , 2008. Work-life balance: a review of the meaning of the balance construct. J Manage Organ 14: 323–327.

[18] Li LZ Bian JY Wang S , 2021. Moving beyond family: unequal burden across mental health patients’ social networks. Qual Life Res (In press). doi: 10.1007/s11136-021-02782-9. 33566303

[19] Wang S Morav L , 2021. Participation in civil society organizations and ethnic minorities’ interethnic friendships in Britain. Br J Sociol (In press). doi: 10.1111/1468-4446.12819. 33751555

[21] He G Chen Y Wang S Dong Y Ju G Chen B , 2020. The association between PM2.5 and depression in China. Dose Response 18: 1–6. 10.1177/1559325820942699 PMC7370340 32733175

[22] Islam MS 2020. COVID-19-Related infodemic and its impact on public health: a global social media analysis. Am J Trop Med Hyg 103: 1621–1629. 32783794 10.4269/ajtmh.20-0812 PMC7543839

[23] Shawky S , 2018. Measuring geographic and wealth inequalities in health distribution as tools for identifying priority health inequalities and the underprivileged populations. Glob Adv Health Med 7: 1–10. 10.1177/2164956118791955 PMC6083744 30109161

[24] Wang S Hu Y , 2019. Migration and health in China: linking sending and host societies. Popul Space Place 25: 22–31.

[25] World Bank , 2020. Population, total (World Bank estimate)—Indonesia. Accessed May 18, 2021. Available at: https://data.worldbank.org/indicator/SP.POP.TOTL?locations=ID.

[26] The COVID-19 National Task Force , 2021. Distribution map. Available at: https://covid19.go.id/peta-sebaran. Accessed May 18, 2021.

[27] Mutia Nasir N Iqbal Nurmansyah M , 2020. Misinformation related to COVID-19 in Indonesia. J Adm Kesehat Indones 8: 51–59.

[28] Wang S Mak H-W , 2020. Generational health improvement or decline? Exploring generational differences of British ethnic minorities in six physical health outcomes. Ethn Health 25: 1041–1054. 29699405 10.1080/13557858.2018.1469736

[29] Heymann DL Shindo N , 2020. COVID-19: what is next for public health? Lancet 395: 542–545. 32061313 10.1016/S0140-6736(20)30374-3 PMC7138015

[30] Wang S Coutts A Burchell B Kamerāde D Balderson U , 2020. Can active labour market programmes emulate the mental health benefits of regular paid employment? Longitudinal evidence from the United Kingdom. Work Employ Soc. 35: 545–565.

[31] Firouzbakht M Omidvar S Firouzbakht S Asadi-Amoli A, 2021. COVID-19 preventive behaviors and influencing factors in the Iranian population; a web-based survey. BMC Public Health 21: 143. 33451303 10.1186/s12889-021-10201-4 PMC7809636

[32] Handayani N Kusumawati A Indraswari R, 2021. Prevention of COVID-19 among Indonesian Moslem. Ann Trop Med Public Health 24. doi: 10.36295/ASRO.2021.24128.

[33] Kim HK Ahn J Atkinson L Kahlor LA , 2020. Effects of COVID-19 misinformation on information seeking, avoidance, and processing: a multicountry comparative study. Sci Commun 42: 586–615. 10.1177/1075547020959670 PMC7492825 38603002

[34] Pennycook G McPhetres J Zhang Y Lu JG Rand DG , 2020. Fighting COVID-19 misinformation on social media: experimental evidence for a scalable accuracy-nudge intervention. Psychol Sci 31: 770–780. 32603243 10.1177/0956797620939054 PMC7366427

[35] Malfait S Eeckloo K Van Hecke A , 2017. The influence of nurses’ demographics on patient participation in hospitals: a cross-sectional study. Worldviews Evid Based Nurs 14: 455–462. 28841757 10.1111/wvn.12254

[36] Agley J Xiao Y Thompson EE Golzarri-Arroyo L , 2020. COVID-19 misinformation prophylaxis: protocol for a randomized trial of a brief informational intervention. JMIR Res Protoc 9: e24383. 33175694 10.2196/24383 PMC7722482

[37] Chou W-YS Oh A Klein WMP , 2018. Addressing health-related misinformation on social media. JAMA 320: 2417–2418. 30428002 10.1001/jama.2018.16865

