# Supplementary material for: Hand Hygiene and Mask-Wearing Practices during COVID-19 among Healthcare Workers: Misinformation as a Predictor
Source: Am J Trop Med Hyg. 2021 Oct 22;105(6):1483–9. doi: 10.4269/ajtmh.21-0463 (PMC8641349; doi:10.4269/ajtmh.21-0463)
Supplement: Supplementary file 1 [file tpmd210463.SD1.pdf]

Supplemental Appendix

Table A1. Ordinary Least Squares (OLS) models examining the associations between COVID misinformation beliefs and frequencies of health preventive behaviors.

**Panel A: Frequency of washing hands**	Model 1	Model 2	Model 3	Model 4
Misinformation beliefs (Ref. = Completely agree or agree)	COVID engineered	COVID likes a common cold	The 5G network spreads COVID	Eating garlic prevents COVID
Neither agree or disagree	-0.45**	-0.26	-0.22	-0.08
	(-0.74 - -0.16)	(-0.57 - 0.06)	(-0.55 - 0.11)	(-0.34 - 0.18)
Disagree or completely disagree	-0.45**	-0.48***	-0.82***	-0.49**
	(-0.74 - -0.16)	(-0.76 - -0.20)	(-1.15 - -0.50)	(-0.81 - -0.17)
Constant	6.13***	6.06***	6.20***	5.89***
	(5.42 - 6.83)	(5.36 - 6.77)	(5.51 - 6.90)	(5.19 - 6.59)
Observations	518	518	518	518
R-squared	0.09	0.09	0.11	0.08
**Panel B: Frequency of wearing a mask**	Model 1	Model 2	Model 3	Model 4
Misinformation beliefs (Ref. = Completely agree or agree)	COVID engineered	COVID likes a common cold	The 5G network spreads COVID	Eating garlic prevents COVID
Neither agree or disagree	-0.28**	-0.27**	-0.03	-0.06
	(-0.46 - -0.11)	(-0.46 - -0.08)	(-0.22 - 0.17)	(-0.22 - 0.09)
Disagree or completely disagree	-0.32***	-0.31***	-0.60***	-0.47***
	(-0.49 - -0.14)	(-0.48 - -0.15)	(-0.79 - -0.40)	(-0.66 - -0.27)
Constant	7.10***	7.08***	7.15***	6.96***
	(6.68 - 7.52)	(6.66 - 7.50)	(6.74 - 7.56)	(6.54 - 7.37)
Observations	518	518	518	518
R-squared	0.12	0.12	0.15	0.13

Note. All models control for age, ethnicity, educational levels, professions, whether have chronic diseases and whether have had flu-like symptoms in the last six months. Confidence intervals are in parentheses. *** p<0.001, ** p<0.01, * p<0.05 (two-tailed tests).
